# Intra- and Inter-Rater Reliability of Three Measurements for Assessing Tactile Acuity in Individuals with Chronic Low Back Pain

**DOI:** 10.1155/2020/8367095

**Published:** 2020-11-24

**Authors:** Juan Wang, Changcheng Chen, Mengsi Peng, Yizu Wang, Bao Wu, Yili Zheng, Xueqiang Wang

**Affiliations:** ^1^Department of Sport Rehabilitation, Shanghai University of Sport, 309 Changhai RD, Shanghai 200438, China; ^2^Department of Rehabilitation Medicine, Shanghai Shangti Orthopaedic Hospital, 188 Hengren RD, Shanghai 200438, China

## Abstract

**Objective:**

To investigate the intra- and inter-rater reliability of three measurements on painful and pain-free sides in participants with chronic low back pain (CLBP) at different ages.

**Methods:**

We recruited 60 participants with CLBP and divided them equally into a group of younger participants with chronic low back pain (18 ≤ age ≤ 35, Y-CLBP) and a group of older participants with chronic low back pain (36 ≤ age ≤ 65, O-CLBP). Participants were assessed by two testers within the same day (10 min interval), and one of the testers repeated the assessment program 24 h later. The intraclass correlation coefficient (ICC) was used to assess reliability. The Pearson correlation coefficient was used to analyze the correlation between tactile acuity and age, waistline, and pain-related variables.

**Results:**

In the Y-CLBP group, the intra-rater reliability of two-point discrimination (TPD), point-to-point test (PTP), and two-point estimation (TPE) on the painful and pain-free sides was good (ICC range: 0.74–0.85), whereas the inter-rater reliability of TPD, PTP, and TPE on the painful and pain-free sides was moderate to good (ICC range: 0.65–0.76). In the O-CLBP group, the intra-rater reliability of TPD, PTP, and TPE on the painful and pain-free sides was good (ICC range: 0.75–0.85), whereas the inter-rater reliability of TPD, PTP, and TPE on the painful and pain-free sides was moderate to good (ICC range: 0.70–0.85). Age, waistline, duration of pain, maximum pain, general pain, and unpleasant score caused by pain were positively correlated with tactile acuity thresholds (D-TPD, A-TPD, PTP, and TPE) (*r* > 0.365, *p* < 0.05). When BMI was controlled, age, waistline, and pain-related variables were positively correlated with tactile acuity thresholds (r > 0.388; *p* < 0.05).

**Conclusion:**

In the participants of Y-CLBP and O-CLBP groups, TPD, PTP, and TPE have moderate-to-good intra- and inter-rater reliability on the painful and pain-free sides of the fifth lumbar vertebrae.

## 1. Introduction

Chronic low back pain (CLBP) is related to the structural and functional cortical changes in some brain regions [1], including primary and secondary cortical reorganization [2, 3]. The tactile acuity threshold is related to the integrity of the primary somatosensory cortex (S1) [4, 5]. In patients with CLBP, the cortical shift of the back is about 2 cm [2]. Patients with complex regional pain syndrome or phantom limb pain exhibit changes in the S1 expression and two-point discrimination (TPD) threshold [6, 7]. With the decrease in pain intensity, somatosensory representation tends to be normalized [8]. Normalization of cortical reorganization may be associated with pain in patients with chronic pain [9]. Whether or not cortical reorganization and tactile acuity defects are the characteristics of all pain conditions remains unclear. Tactile acuity training is a feasible target for the treatment of pain. It can effectively relieve pain and improve function [10], which lays a foundation for the research of cortical reorganization.

Cortical reorganization in patients with CLBP has been confirmed [2], and the research on cortical reorganization is deepening [10, 11]. Measuring cortical remodeling reliably and effectively is necessary for clinical assessment and intervention. Electroencephalography and functional magnetic resonance imaging are the gold standard methods for measuring cortical remodeling [1, 12, 13]. These methods require sophisticated technology, are time intensive, and are of high cost. Therefore, evaluation methods that are cheap, portable, and easy to operate have been developed in a clinic. TPD is a simple clinical tactile acuity test for measuring the minimum distance between two perceived points on the skin [14]. It was originally used for the tactile acuity examination of hands and fingers [15]. Recent studies have used TPD to assess lower back tactile acuity [16]. The point-to-point test (PTP) is used to measure the distance between the location point and the perception point on the participant's skin. In addition, the PTP has been widely used in the study of lower back tactile acuity [17]. Two-point estimation (TPE) compares the actual distance between two stimulus points with the estimated distance of the participant. Compared with PTP, TPE is unaffected by upper limb mobility because it does not need upper limb rotation and posterior extension to point out stimulus points during the test. Hence, TPE is also used to evaluate lower back tactile acuity [18].

So far, the differences in tactile acuity between painful and pain-free areas of the body have only been confirmed in a few diseases, such as chronic neck pain [19]. Although CLBP is a leading cause of disability worldwide, the differences in tactile acuity between the painful and pain-free sides of the fifth lumbar vertebrae (L5) in patients with CLBP remain unknown [20]. In addition, low back pain can occur in low-, medium-, and high-income countries and all ages from children to the elderly [21]. Age is another major factor affecting tactile acuity [22]. Growing evidence indicates that the processing capacity of the central cortex changes with age, which leads to changes in the composition of skin receptors [23]. In healthy adults, tactile acuity declines at the age of over 35 [24]. Age [25] and pain [26] are important factors affecting cortical reorganization. Therefore, this study has the following objectives: (1) to explore and confirm the intra- and inter-rater reliability of TPD, PTP, and TPE in the painful and pain-free sides of participants with CLBP at different ages; (2) to compare the tactile acuity thresholds of the painful and pain-free sides of participants with CLBP at different ages; and (3) to explore the correlations between tactile acuity and age, waistline, and pain-related variables.

## 2. Materials and Methods

### 2.1. Study Design

In this study, 60 participants with CLBP underwent the same TPD, PTP, and TPE tests conducted by two testers at three time points to estimate their lumbar tactile acuity. To obtain the reliability of the lumbar tactile acuity, the results of the same tester's pre- and post-test (24 h interval) were compared to verify the intra-rater reliability, whereas the results of the two testers were compared (10 min interval) to verify the inter-rater reliability. Two examiners were blinded about the disease state and about which side of the L5 was painful. The characteristics of all participants were recorded by another examiner before measurement.

### 2.2. Participants

With reference to a method described by one study [27], the sample size was decided that for three repeated measurements of TPD, PTP, and TPE for each person and *P*1 (intraclass correlation coefficient, ICC) = 0.8, *P*0 (ICC) = 0.6, with hypothesis of *α* = 0.05 and 80% power, 27 participants were needed for reliability analysis. According to the age of participants with CLBP, a group of younger participants with CLBP (18 ≤ age ≤ 35, Y-CLBP) and a group of older participants with CLBP (36 ≤ age ≤ 65, O-CLBP) were recruited, with 30 people in each group and 60 people in total.

Sixty-five participants with CLBP were enrolled in this study. After excluding two participants with a history of upper limb injury and three participants with bilateral low back pain, 60 participants with CLBP (29 men and 31 women) were finally included. The participants were divided into a young CLBP group (Y-CLBP, 18 ≤ age ≤ 35) and an old CLBP group (O-CLBP, 36 ≤ age ≤ 65).

The inclusion and exclusion criteria of the participants were derived from the study of Ehrenbrusthoff et al. [28]. Participants were included if (1) they were between 18 and 65 years old; (2) the duration of their CLBP symptoms has lasted more than 6 months, and they have persistent CLBP ≥ 50% of the time in the past 6 months; (3) they experience unilateral lumbar pain, with the pain area including the L5; and (4) they have no damage to the shoulders, elbows, and wrists, and their range of motion was normal. Participants were excluded in this study if (1) they have a mental or cognitive impairment, (2) they were incapable of understanding and executing oral or written instructions, or (3) they had a history of spinal surgery. All participants were informed of the content and purpose of the experiment and signed a written informed consent form before the test.

### 2.3. Preparation

**Figure 1:**
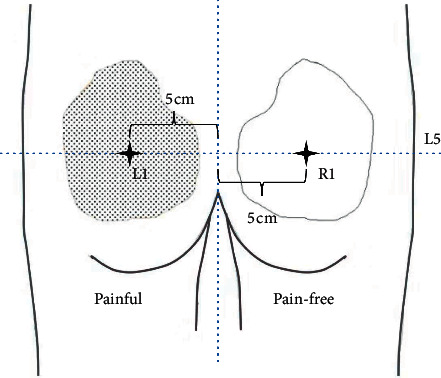
Positioning of measurement points. Localization of measurement sites. The painful area (for example, the left side) was outlined with a pen and copied onto pain-free areas (for example, the right side). The black cross refers to the point about 5 cm from the vertical distance of the L5 spinous process. The TPD and TPE tasks were performed in such a way that the caliper's tips covered the marked point, and the PTP task was performed in such a way that the caliper's tip stimulus marker points, painful, and pain-free areas were complementary to each other.

Participants were comfortably prone on the treatment bed with their lower back exposed [29, 30]. Their upper limbs were naturally placed vertically on both sides of the treatment bed. The lower back was relaxed, and the muscles were not contracted. According to the standardized palpation method in previous studies [31], the examiner located the L5 spinous process and marked it with a black pen, drew the horizontal axis at the L5 spinous process level, and then drew two points on the left and right sides 5 cm from the midline. Tactile acuity was measured at these two points (Figure 1). This standardization refers to the scheme described in previous studies [16, 28].

### 2.4. Tactile Acuity Assessment

TPD, PTP, and TPE were measured using a mechanical sliding caliper (Powerfix, digital caliper: Z22855) with a measurement accuracy of 0.01 mm. The order of the test procedure and the examiner was randomized.

TPD is a simple clinical tactile acuity test for measuring the minimum distance between two perceived points on the skin [14]. The TPD test was evolved from the previous TPD threshold measurement protocol [30]. It was measured along the L5 horizontal line (1R or 1L) with reference to previous research methods [17]. First, TPD was carried out in an ascending manner (A-TPD). The calipers were used on the premarked line until the first time they blanched the skin [15]. They were used to stimulate the skin along the L5 horizontal line and the waist curve as much as possible so that the two stimulating points reach the skin at the same time but not in tandem. Testing started with a 20 mm range between the two tips of the caliper and widened in 5 mm increments until the participants could verbally report that they have touched two points instead of one point. If the participant initially identified that the stimulus was two points, then the stimulus distance was increased by 5 mm. If the participant noticed two points again, then such circumstance was deemed to represent the conformity of the identification, and the first distance identified by the two points was treated as the TPD result. This procedure was based on a preliminary preview of the process. In the pre-experiment, 1 mm increments were time-consuming, and participants reported that excessive stimulation easily numbed the skin. Moreover, the ranges between 0 and 20 mm were repeatedly confirmed as one point. Afterwards, TPD was carried out in descending order (D-TPD) until one point was perceived. The starting distance of the caliper in the descending series is the distance between two points perceived by the patient in the ascending series. The above process was repeated three times. Hence, the TPD score consisted of an average of six staircases, three increases and three decreases. Lower values demonstrated better tactile acuity.

The PTP is used to measure the distance between the location point and the perception point on the participant's skin. In recent years, the PTP has been used to study lower back tactile acuity [17]. The PTP test was carried out based on a previously published study [17]. The tester gently touched one of the points (1R or 1L), and the participants were asked to mark stimulus points with a pen in the prone position. The distance between the touched and marked points was measured using calipers for the PTP. The average of three repeated tests was taken as the PTP score. Lower PTP values demonstrated better tactile acuity.

TPE compares the actual distance between two stimulus points with the estimated distance of the participant. Two calipers were used in the TPE test; one for the tester and one for the participant. The tester applied a tactile stimulus along the L5 horizontal line until the first time it blanched the skin with a horizontal interval of 120 mm [18, 30, 32] between the tips of the caliper. The participants were asked to manually indicate their perceived distance with their calipers. The participants held the caliper and were blinded to the scale and digital display on the back of the caliper. Each tester repeated the measurements thrice, and the mean was calculated.

### 2.5. Participants' Profile

Before the test, the participants were asked to report their current pain duration, general pain intensity, pain unpleasantness, and maximum pain in the last 3 months. They used a 10-point numerical rating scale to express pain intensity and unpleasantness, with “0” indicating no pain/no unpleasantness and “10” indicating the most pain/the most unpleasantness. Pain duration was assessed in months. In addition, the Oswestry Disability Index (ODI) and the Roland–Morris Disability Questionnaire (RMDQ) were used to evaluate the function and disability of the participants.

### 2.6. Statistical Analysis

All results were analyzed using SPSS, version 20.0 (SPSS 3 Inc., Chicago, IL, USA). The ICC model (3, k) and the ICC model (2, k) were used in the intra-rater and inter-rater reliability analyses, respectively. Different guidelines for classifying the ICC value, which is a reasonable scale for expressing the grade of reliability, were used as follows: >0.90, excellent; 0.75–0.90, good; 0.50–0.75, moderate; and <0.50, poor [33]. Accuracy of the assessment methods was evaluated by the standard error of measurement (SEM) for the intra- and inter-rater reliability via the following formula [34, 35]: SEM = SD × $\sqrt{1-\text{ICC}}$, where SD is the standard deviation of the measured results, and ICC is the reliability coefficient of the data. Furthermore, SD could represent the degree of spread of a data set. The level of variation of data will vary with the change in SD. Finally, the result values were rounded to two decimal points and then noted down. The tactile acuity thresholds of the painful and pain-free sides between the Y-CLBP and O-CLBP groups were compared using the independent-sample *t*-test. To explore the influence of related variables on tactile acuity scores, we randomly selected the measurement results of examiner A or B and used the Pearson correlation coefficient to analyze the correlation between tactile acuity and age, waistline, pain duration, maximum pain, general pain, and pain unpleasantness. In addition, we used partial correlation analysis to determine the correlation between age waistline, pain-related variables, and tactile acuity when controlling for BMI. The magnitude of correlation is expressed by the coefficient *r*. The standard of statistical significance of all tests was set as *p* < 0.05.

In this experiment, the Bland–Altman plot was used to visualize the numerical distribution. From this analysis, the difference between the two testers or between two sessions of assessments was depicted against their average value. A total 95% of the discrepancy was assumed to be within the limits of agreement (LOA).

## 3. Results

Table 1 shows the profiles and descriptive statistics of the participants. Sixty participants were recruited, including 30 participants of Y-CLBP (15 females) and 30 participants of O-CLBP (16 females). No significant differences in height and weight were found between groups (*p* > 0.05). Compared with the Y-CLBP group, the O-CLBP group showed significantly higher age, body mass index, pain duration, maximum pain, general pain, pain unpleasantness (using the digital rating scale), ODI, and RMDQ scores (*p* < 0.05).  Table 2 shows the raw mean and SDs of TPD, PTP, and TPE.  Table 3 shows the *p* value of the tactile acuity threshold comparison between the painful and pain-free sides in the Y-CLBP and O-CLBP groups. As shown in Tables 2 and 3, the threshold of tactile acuity in the O-CLBP group was higher than that in the Y-CLBP group (difference range: 1.97–25.92 mm), and the tactile acuity threshold of the painful side was higher than that of the pain-free side (difference range: 0.2–13.21 mm), regardless of the examiner, TPD, PTP, or TPE variables, and most of the differences were statistically significant (*p* value range = 0–0.043, *p* < 0.05). In the Y-CLBP and O-CLBP groups, the number of test items with significant difference in the tactile acuity threshold between the painful and pain-free sides was the same (i.e., 8). Nine test items had significant difference in the tactile acuity threshold on the painful and pain-free sides between the two groups.

Tables 4 and 5 show the intra-rater and inter-rater reliability data, respectively, along with each session's ICC, 95% confidence interval (CI), mean difference, SD, and SEM for each group. For the sake of simplicity of data reporting, the reliability coefficient was calculated by using the mean of three measurement results. The three measurements of the Y-CLBP and O-CLBP groups showed good intra-rater reliability and moderate-to-good inter-rater reliability on the painful and pain-free sides, respectively. In the Y-CLBP group, the intra-rater reliability of D-TPD, A-TPD, PTP, and TPE on the painful and pain-free sides was good, whereas the inter-rater reliability of D-TPD, A-TPD, PTP, and TPE on the painful and pain-free sides was moderate to good. In the O-CLBP group, the intra-rater reliability of D-TPD, A-TPD, PTP, and TPE on the painful and pain-free sides was good, whereas the inter-rater reliability of D-TPD, A-TPD, PTP, and TPE on the painful and pain-free sides was moderate to good.

Considering that no significant difference in tactile acuity scores was measured by examiners A and B, we randomly selected one data set to perform e PTP test increased, and the distance estimation between the t the Pearson correlation coefficient analysis and partial correlation analysis (Tables 6 and 7). Age, waistline, pain duration, maximum pain, general pain, and pain unpleasantness were positively correlated with the tactile acuity (D-TPD, A-TPD, PTP, and TPE) threshold (*r* > 0.365, *p* < 0.05). When BMI was controlled, age, waistline, and pain-related variables were positively correlated with tactile acuity thresholds (r > 0.388; p < 0.05).

**Figure 2:**
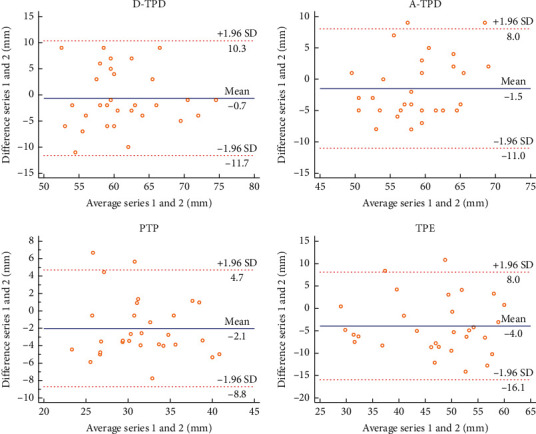
Mean difference between the first and second series of measurements plotted against the average scores of TPD (D-TPD and A-TPD), PTP, and TPE on the painful side of the Y-CLBP group.

**Figure 3:**
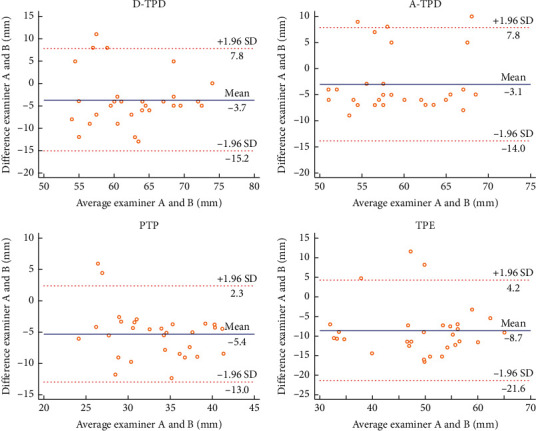
Mean difference between two examiners plotted against the average scores of TPD (D-TPD and A-TPD), PTP, and TPE on the painful side of the Y-CLBP group.

**Figure 4:**
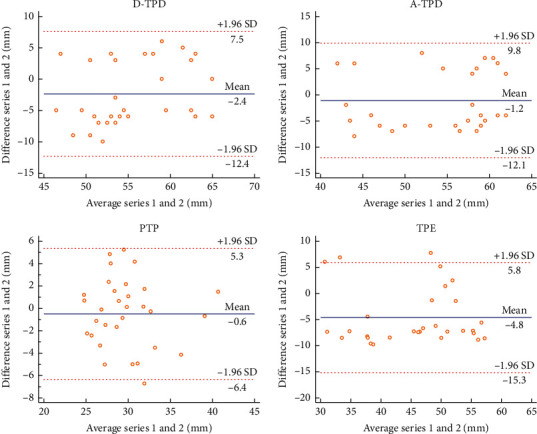
Mean difference between the first and second series of measurements plotted against the average scores of TPD (D-TPD and A-TPD), PTP, and TPE on the pain-free side of the Y-CLBP group.

**Figure 5:**
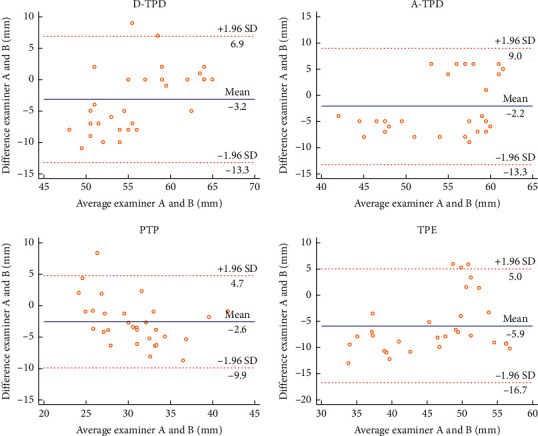
Mean difference between two examiners plotted against the average scores of TPD (D-TPD and A-TPD), PTP, and TPE on the pain-free side of the Y-CLBP group.

**Figure 6:**
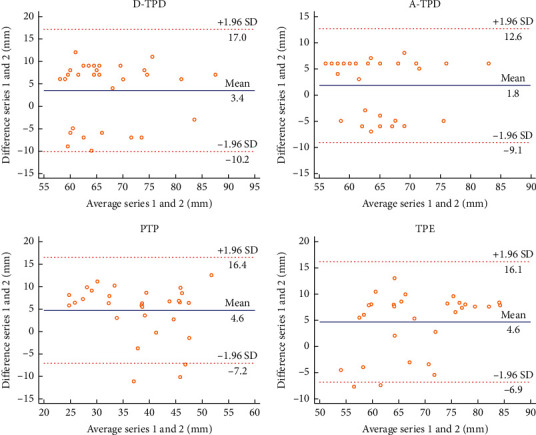
Mean difference between the first and second series of measurements plotted against the average scores of TPD (D-TPD and A-TPD), PTP, and TPE on the painful side of the O-CLBP group.

**Figure 7 fig7:**
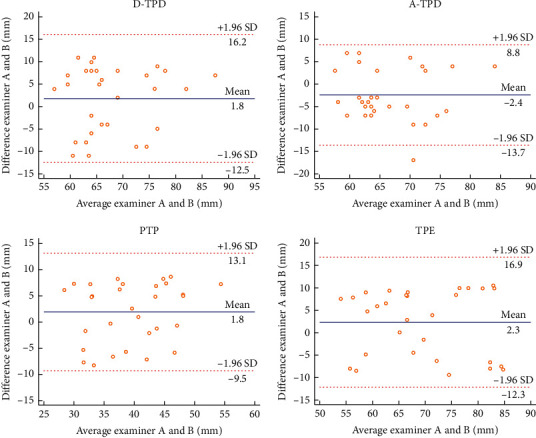
Mean difference between two examiners plotted against the average scores of TPD (D-TPD and A-TPD), PTP, and TPE on the painful side of the O-CLBP group.

**Figure 8 fig8:**
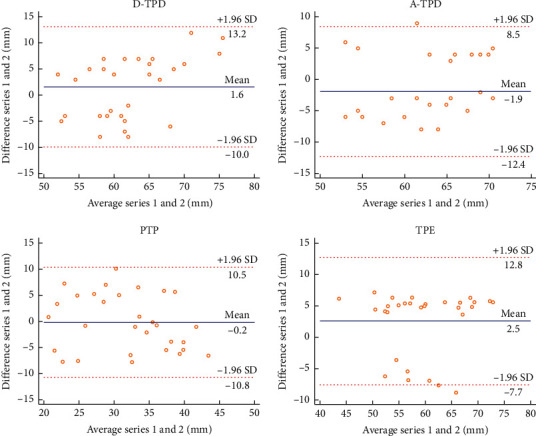
Mean difference between the first and second series of measurements plotted against the average scores of TPD (D-TPD and A-TPD), PTP, and TPE on the pain-free side of the O-CLBP group.

**Figure 9:**
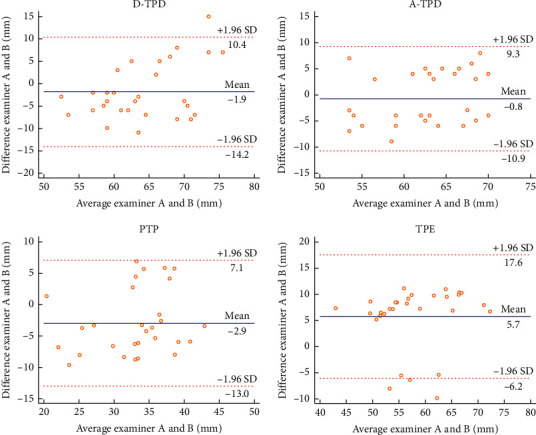
Mean difference between two examiners plotted against the average scores of TPD (D-TPD and A-TPD), PTP, and TPE on the pain-free side of the O-CLBP group.

The Bland–Altman plot confirmed the acceptable LOA between the testers and the sessions, showing no systematic deviation (Figures 2–9). Most of the differences (95%) were spread within the LOA (difference ± 1.96 SD). In the Y-CLBP group, the intra-rater ICC range of the three measurement methods on the painful and pain-free sides was 0.74–0.85 (Figures 2 and 4); the inter-rater ICC range of the three measurement methods on the painful and pain-free sides was 0.65–0.76 (Figures 3 and 5). In the O-CLBP group, the intra-rater ICC range of the three measurement methods on the painful and pain-free sides was 0.75–0.85 (Figures 6 and 8), whereas the inter-rater ICC range of the three measurement methods on the painful and pain-free sides was 0.65–0.76 (Figures 7 and 9).

## 4. Discussion

Results showed that the intra- and inter-rater reliability of the three measurement methods in the Y-CLBP and O-CLBP groups were good and moderate to good, respectively. The threshold of tactile acuity on the painful side was higher than that on the pain-free side, and the tactile acuity threshold of the O-LBP group was higher than that of the Y-CLBP group.

Three previous studies have reported the intra- and inter-rater reliability of the lumbar TPD. Catley et al. [29] reported that 28 doctors assessed the TPD of L3 spinal level in 28 healthy young people and found that the intra-rater ICC was 0.81 and the inter-rater ICC was 0.66. Adamczyk et al. [17] indicated that two testers assessed the TPD of L3 spinal level in 21 healthy young people and found that the intra-rater ICC was 0.72 and the inter-rater ICC was 0.56. Ehrenbrusthoff et al. [28] reported that two testers assessed the TPD of the L5 spinal level in 35 s with nonspecific CLBP (NCLBP), and three repeated measurements revealed that the intra-rater ICC was 0.8 and the inter-rater ICC was 0.53. In our study, including the painful and pain-free sides, the intra-rater ICC ranges of TPD in the Y-CLBP and O-CLPB groups were 0.74–0.82 and 0.75–0.81, respectively, and the inter-rater ICC ranges of TPD in the Y-CLBP and O-CLPB groups were 0.66–0.75 and 0.72–0.78, respectively. Overall, the intra- and inter-rater ICC ranges of TPD were similar between studies, although some differences may be caused by differences in measurement locations, measurement intervals, and populations. Only one study confirmed the reliability of PTP in healthy people [17]; this study assessed the PTP at three locations on the L3 level and found that the PTP reliability at the location 5 cm away from the L3 spinous process was the highest. In the above study [17], the inter-rater ICC was 0.86 (measured at 10 min interval), and the intra-rater ICC values were 0.84 (measured at a 10-min interval) and 0.85 (measured at a 1-week interval). Our study found that the intra- and inter-rater ICC ranges of PTP were 0.77–0.85 and 0.65–0.80, respectively. The difference between the two studies is that the measurement sites were L3 and L5, and the participants were healthy people and participants with CLBP, respectively. In addition, the age of healthy participants is similar to that of participants with Y-CLBP, but the PTP threshold of the former is lower than that of the latter. The difference in PTP thresholds between two studies may be due to the so-called expand problem in whole body perception of patients with CLBP [36, 37], which leads to overestimation of trunk contour and two-point spacing.

Luedtke et al. [38] studied the reliability of two versions of TPE (manual and oral) for the most painful and pain-free sides of the lower back. The inter-rater reliability of the manual version (ICC = 0.75–0.91) was higher than that of the oral version (ICC = 0.53–0.88). Moreover, the intra-rater reliability (interval period was 2 days) of the manual version (ICC = 0.75–0.91) was higher than that of the oral version (ICC = 0.67–0.84). Similarly, in our study, the intra-rater reliability of the manual version was 0.82–0.85, and the inter-rater reliability was 0.70–0.85. The difference is that we tested the TPE reliability of the painful (not the most painful area) and pain-free sides.

Previous studies have confirmed the reliability of TPD in healthy people and patients with NCLBP [28, 29], PTP in healthy people [17], and TPE in patients with CLBP [38]. Our study confirmed that TPD, PTP, and TPE tests had good intra-rater reliability and moderate-to-good inter-rater reliability in participants with CLBP at different ages. Therefore, TPD, PTP, and TPE tests can be extended to the evaluation of lumbar tactile acuity in different populations and ages. The present study also found that the tactile acuity threshold of older people (36–65 years) was higher than that of younger people (18–35 years), and the tactile acuity threshold of the painful side was higher than that of the pain-free side, which coincided with the previous findings that age [39] and pain [40] affect tactile acuity. Previous studies have suggested that patients with CLBP perceive the expanded or shrunken back image, which is called the contract-expand problem [36, 37, 41]. Moseley [37] found that five out of six CLBP patients cannot draw the outline of their trunk, which is related to the larger TPD threshold. This qualitative observation has been more quantitatively demonstrated in our research.

The Pearson correlation coefficient showed that age, waistline, pain duration, maximum pain, general pain, and pain unpleasantness were positively correlated with TPD, PTP, and TPE scores. This observation shows that as the age, waistline, pain duration, maximum pain, general pain, and pain unpleasantness increase, the threshold of TPD and the distance between the stimulation point and perception point in the PTP test increased, and the distance estimation between the two tactile stimuli decreased. When BMI was controlled, age, waistline, and pain-related variables were positively correlated with tactile acuity thresholds (r > 0.388; p < 0.05). This observation shows that age, waistline, and pain-related variables were positively correlated with tactile acuity. Previous studies have shown that pain-related variables influence TPE [18]. The TPD threshold of healthy people is independently related to age, and the effect of age on the TPD threshold is significantly affected by the waist-to-hip ratio [39]. The effect of pain on tactile acuity may be related to the relationship between the tactile acuity threshold and the integrity of S1 [4, 5]. Patients with CLBP have primary and secondary cortical reorganization. An obvious clinical feature of altered S1 expression is the change in tactile acuity [6, 7]. Previous studies have proved that tactile acuity training can effectively relieve the pain [42, 43] of patients with CLBP, normalize the cortical reorganization [10], and improve tactile acuity [44].

### 4.1. Strengths and Limitations

In spite of the effective reliability of the three tests for tactile acuity (TPD, PTP, and TPE), their validity types need further study. In this experiment, the age group of the participants was limited to 18–65 years; in the future, the age group should be enlarged so that the three tests can be applied to more people. Another limitation is that our evaluation program is executed in a prone position. While this method refers to other protocols, it may aggravate the pain for some participants with CLBP. Future research could investigate the measurement characteristics of this evaluation program in the participant's comfort position. In addition, body hair, age, skin temperature, tiredness, attention, collaboration, and stimulus strength are important factors affecting tactile acuity measurement [29, 45–47].

The primary advantage of the present study is its focus on participants with CLBP of different ages and its analysis on the intra- and inter-rater reliability for the three assessment methods on their painful and pain-free sides. Another advantage of this study is the comparison of tactile acuity thresholds on the painful and pain-free sides at different ages. The study systematically reflected the reliability of three assessment methods on both sides of the L5 level in participants with CLBP as well as divided the age group and painful side of the participants to determine the effects of age and pain on tactile acuity accurately. The three assessment methods will hopefully be extended to more patients with CLBP and applied to more body regions.

## 5. Conclusion

In the Y-CLBP and O-CLBP groups, TPD, PTP, and TPE are reliable methods for measuring the tactile acuity of painful and pain-free sides at the L5 level. The reliability (ICC ≥ 0.65) of the three measurements is sufficient to assess the tactile acuity of participants with CLBP in future studies.

## Figures and Tables

**Table 1:** Participant characteristics.

	Y-CLBP (18–35 yr)	O-CLBP (36–65 yr)	Total
*n* (%)	30 (50%)	30 (50%)	60 (100%)
Age (years)	25.23 ± 4.12	50.00 (10.47)	36.80 (15.29)
Height (cm)	170.17 ± 8.45	168.01 (7.66)	168.84 (8.08)
Weight (kg)	64.85 ± 9.62	67.59 (11.44)	66.07 (10.72)
BMI (kg/m^2^)	22.35 ± 2.66	23.95 (2.43)	23.15 (2.66)
Waistline (cm)	74.75 (4.76)	102.95 (6.23)	88.85 (15.24)
Sex, no. of females (%)	15/30 (50%)	16/30 (52%)	31 (52%)
Pain duration, month	25.50 (14.65)	93.97 (22.71)	59.73 (39.38)

NRS			
Maximum pain	4.27 (1.08)	6.07 (0.74)	5.17 (1.29)
General pain	3.13 (1.01)	5.00 (0.79)	4.07 (1.30)
Pain unpleasantness	5.70 (0.88)	7.43 (0.86)	6.57 (1.23)
ODI	15.27 (4.46)	27.33 (4.82)	21.30 (7.63)
RMDQ	4.33 (1.09)	9.8 (2.71)	7.07 (3.43)

^*∗*^Values as mean units (±standard deviation); Y-CLBP = younger participants with chronic low back pain; O-CLBP = older participants with chronic low back pain; BMI: body mass index. NRS = Numerical Rating Scale; ODI = Oswestry Disability Index; RMDQ = Roland–Morris Disability Questionnaire; SAS = self-rating anxiety scale; SDS = Self-Rating Depression Scale.

**Table 2:** Raw tactile acuity scores collected by examiners A and B.

Group	Method	Order	Examiner A					Examiner B				
			Painful side		Pain-free side		Painful side − pain-free side	Painful side		Pain-free side		Painful side − pain-free side
			Mean	SD	Mean	SD	Mean	Mean	SD	Mean	SD	Mean
Y-CLBP	D-TPD	1	60.77	6.22	54.27	6.64	6.50	64.47	6.47	57.47	4.07	7.00
		2	61.43	6.23	56.70	5.24	4.73					
	A-TPD	1	58.07	6.34	53.83	7.57	4.23	61.17	6.00	56.00	5.40	5.17
		2	59.57	5.06	55.00	6.78	4.57					
	PTP	1	30.59	4.76	29.44	3.98	1.15	35.97	5.82	32.04	5.47	3.93
		2	32.64	5.14	30.02	4.26	2.62					
	TPE	1	44.67	9.68	43.17	8.55	1.50	53.37	9.95	49.03	6.65	4.34
		2	48.69	10.11	47.95	8.87	0.74					

O-CLBP	D-TPD	1	68.73	8.56	63.10	7.95	5.63	66.90	7.88	65.03	5.49	1.87
		2	65.33	8.23	61.53	5.36	3.80					
	A-TPD	1	65.20	6.93	61.70	6.74	3.50	67.63	6.98	62.53	5.16	5.10
		2	63.63	6.26	63.43	7.20	0.20					
	PTP	1	40.42	7.67	31.82	6.58	8.60	38.62	6.64	34.77	7.75	3.85
		2	35.78	9.10	32.00	7.74	3.78					
	TPE	1	70.59	10.15	60.78	7.87	9.81	68.31	10.85	55.10	7.35	13.21
		2	65.99	8.14	58.24	7.69	7.75					

O-CLBP − Y-CLBP	D-TPD	1	7.97		8.83			2.43		7.57		
		2	3.90		4.83							
	A-TPD	1	7.13		7.87			6.47		6.53		
		2	3.87		8.63							
	PTP	1	9.83		2.38			2.65		2.73		
		2	3.14		1.97							
	TPE	1	25.92		17.61			14.94		6.07		
		2	17.30		10.29							

Data presented in the table are derived from the first session (day 1) by examiners A and B and the second session (day 2). SD = standard deviation; Y-CLBP = younger participants with chronic low back pain; O-CLBP = older participants with chronic low back pain; O-CLBP − Y-CLBP = the tactile acuity threshold of the O-CLBP group minus the tactile acuity threshold of the Y-CLBP group; D-TPD = two-point discrimination test was carried out in a descending manner; A-TPD = two-point discrimination test was carried out in an ascending manner; PTP = point-to-point test; TPE = two-point estimation; Painful side − pain-free side = the tactile acuity threshold on painful side minus the tactile acuity threshold on the painful side.

**Table 3:** Comparison of tactile acuity thresholds between painful and pain-free side in two groups.

Examiner	Variable	*P*_Y_	*P*_O_	*P*_YO1_	*P*_YO2_
Examiner A_1_	D-TPD	0.000^*b*^	0.011^*a*^	0.000^*b*^	0.000^*b*^
	A-TPD	0.022^*a*^	0.052	0.000^*b*^	0.000^*b*^
	PTP	0.314	0.000^*b*^	0.000^*b*^	0.097
	TPE	0.527	0.000^*b*^	0.000^*b*^	0.000^*b*^

Examiner A_2_	D-TPD	0.002^*b*^	0.039^*a*^	0.043^*a*^	0.001^*b*^
	A-TPD	0.004^*b*^	0.909	0.020^*a*^	0.000^*b*^
	PTP	0.036^*a*^	0.088	0.107	0.227
	TPE	0.763	0.000^*b*^	0.000^*b*^	0.000^*b*^

Examiner B	D-TPD	0.000^*b*^	0.292	0.197	0.000^*b*^
	A-TPD	0.001^*b*^	0.002^*b*^	0.000^*b*^	0.000^*b*^
	PTP	0.009^*b*^	0.019^*a*^	0.106	0.065
	TPE	0.052	0.000^*b*^	0.000^*b*^	0.001^*b*^

The independent sample *t*-test was used for statistical analysis. ^*a*^: *p* < 0.05; ^*b*^: *p* < 0.01; *P*_Y_: comparison of the tactile acuity thresholds between the painful and pain-free sides in younger participants with chronic low back pain (Y-CLBP); *P*_O_: comparison of the tactile acuity thresholds between the painful and pain-free sides in older participants with chronic low back pain (O-CLBP); *P*_YO1_: comparison of the tactile acuity thresholds on the painful side between the Y-CLBP and O-CLBP groups; *P*_YO2_: comparison of the tactile acuity thresholds on the pain-free side between the Y-CLBP and O-CLBP groups; D-TPD = two-point discrimination test was carried out in a descending manner; A-TPD = two-point discrimination test was carried out in an ascending manner; PTP = point-to-point test; TPE = two-point estimation.

**Table 4:** Intra-rater reliability of rater 1.

Group	Method	Painful side					Pain-free side				
		ICC	95% CI	Mean difference	SD	SEM	ICC	95% CI	Mean difference	SD	SEM
Y-CLBP	D-TPD	0.75	0.48–0.88	0.67	6.18	3.09	0.74	0.45–0.87	2.43	6.06	2.84
	A-TPD	0.77	0.51–0.89	1.50	5.73	2.69	0.82	0.62–0.91	1.67	7.15	3.03
	PTP	0.82	0.63–0.91	2.05	5.02	1.81	0.85	0.68–0.93	0.58	4.09	1.58
	TPE	0.85	0.69–0.93	4.02	10.02	3.32	0.82	0.61–0.91	4.78	8.97	2.84

O-CLBP	D-TPD	0.75	0.48–0.88	3.38	8.50	3.90	0.76	0.49–0.88	1.54	6.77	3.25
	A-TPD	0.81	0.60–0.91	1.77	7.06	3.00	0.78	0.54–0.89	1.95	6.52	2.92
	PTP	0.77	0.52–0.89	4.64	8.66	3.35	0.84	0.66–0.92	0.18	7.12	2.94
	TPE	0.82	0.63–0.91	4.60	9.41	3.12	0.85	0.68–0.93	2.54	7.82	2.82

ICC = intraclass correlation coefficient; CI = confidence interval; mean difference = the difference between the mean of trials 1 and 2; SD = standard deviation; SEM = standard error of measurement; Y-CLBP = younger participants with chronic low back pain; O-CLBP = older participants with chronic low back pain; D-TPD = two-point discrimination test was carried out in a descending manner; A-TPD = two-point discrimination test was carried out in an ascending manner; PTP = point-to-point test; TPE = two-point estimation.

**Table 5:** Inter-rater reliability of rater 1 and rater 2.

Group	Method	Painful side					Pain-free side				
		ICC	95% CI	Mean difference	SD	SEM	ICC	95% CI	Mean difference	SD	SEM
Y-CLBP	D-TPD	0.66	0.22–0.85	3.70	6.57	3.41	0.66	0.22–0.84	3.20	5.69	3.01
	A-TPD	0.70	0.32–0.86	3.10	6.31	3.16	0.75	0.48–0.88	1.00	6.61	3.17
	PTP	0.65	−0.21–0.88	5.38	5.93	2.30	0.76	0.34–0.90	2.60	4.92	2.09
	TPE	0.72	−0.18–0.91	4.68	10.67	3.85	0.73	−0.02–0.90	5.86	8.15	3.16

O-CLBP	D-TPD	0.75	0.48–0.88	1.82	8.21	4.11	0.72	0.42–0.87	1.97	6.84	3.55
	A-TPD	0.77	0.51–0.89	2.43	7.00	3.21	0.78	0.53–0.89	0.85	5.97	2.80
	PTP	0.80	0.58–0.90	1.80	7.17	3.13	0.75	0.40–0.89	2.95	6.30	2.89
	TPE	0.85	0.68–0.93	2.28	10.48	3.92	0.70	0.04–0.89	5.69	8.08	3.52

ICC = intraclass correlation coefficient; CI = confidence interval; mean difference = the difference between the mean of trials 1 and 2; SD = standard deviation; SEM = standard error of measurement; Y-CLBP = younger participants with chronic low back pain; O-CLBP = older participants with chronic low back pain; D-TPD = two-point discrimination test was carried out in a descending manner; A-TPD = two-point discrimination test was carried out in an ascending manner; PTP = point-to-point test; TPE = two-point estimation.

**Table 6:** Correlation coefficient between age, waistline, pain-related variables, and tactile acuity.

Variables	D-TPD	A-TPD	PTP	TPE
Age	0.366 (0.004)	0.428 (0.001)	0.628 (0.000)	0.594 (0.000)
Waistline	0.469 (0.000)	0.483 (0.000)	0.669 (0.000)	0.694 (0.000)
Pain duration	0.419 (0.001)	0.469 (0.000)	0.655 (0.000)	0.635 (0.000)
Maximum pain	0.365 (0.004)	0.411 (0.001)	0.579 (0.000)	0.440 (0.000)
General pain	0.389 (0.002)	0.415 (0.001)	0.610 (0.000)	0.456 (0.000)
Pain unpleasantness	0.366 (0.004)	0.445 (0.000)	0.586 (0.000)	0.455 (0.000)

^*∗*^Values as *r* (P); D-TPD = two-point discrimination test was carried out in a descending manner; A-TPD = two-point discrimination test was carried out in an ascending manner; PTP = point-to-point test; TPE = two-point estimation.

**Table 7:** Correlation coefficient between age, pain related variables and tactile acuity when controlling for BMI.

Variables	D-TPD	A-TPD	PTP	TPE
Age	0.410 (0.001)	0.456 (0.000)	0.615 (0.000)	0.605 (0.000)
Waistline	0.511 (0.000)	0.507 (0.000)	0.657 (0.000)	0.705 (0.000)
Pain duration	0.456 (0.000)	0.491 (0.000)	0.642 (0.000)	0.641 (0.000)
Maximum pain	0.388 (0.002)	0.421 (0.001)	0.561 (0.000)	0.432 (0.001)
General pain	0.413 (0.001)	0.425 (0.001)	0.594 (0.000)	0.448 (0.000)
Pain unpleasantness	0.390 (0.002)	0.457 (0.000)	0.568 (0.000)	0.448 (0.000)

Values as *r* (P), ^*∗*^*p* < 0.05; D-TPD = two-point discrimination test was carried out in a descending manner; A-TPD = two-point discrimination test was carried out in an ascending manner; PTP = point-to- point test; TPE = two-point estimation.

## Data Availability

The data used to support the findings of this study are available from the corresponding author via e-mail for researchers who meet the criteria for access to confidential data.

## Appendix

Figures 2–5 show the mean difference between the first and second measurements of one examiner and the mean difference between the two examiners plotted separately against the average scores of TPD (D-TPD and A-TPD), PTP, and TPE on the painful or pain-free sides of the Y-CLBP group. The solid horizontal line illustrates the mean difference value with 95% limits of agreement (dotted lines).

Figure 6 [Fig fig7] [Fig fig8]–9 show the mean difference between the first and second measurements of one examiner and the mean difference between the two examiners plotted separately against the average scores of TPD (D-TPD and A-TPD), PTP, and TPE on the painful or pain-free sides of O-CLBP. The solid horizontal line illustrates the mean difference value with 95% limits of agreement (dotted lines).

## References

[1] Wand B. M., Parkitny L., O’Connell N. E. (2011). Cortical changes in chronic low back pain: current state of the art and implications for clinical practice. *Manual Therapy*.

[2] Flor H., Braun C., Elbert T., Birbaumer N. (1997). Extensive reorganization of primary somatosensory cortex in chronic back pain patients. *Neuroscience Letters*.

[3] Hotz-Boendermaker S., Marcar V. L., Meier M. L., Boendermaker B., Humphreys B. K. (2016). Reorganization in secondary somatosensory cortex in chronic low back pain patients. *Spine*.

[4] Duncan R. O., Boynton G. M. (2007). Tactile hyperacuity thresholds correlate with finger maps in primary somatosensory cortex (S1). *Cerebral Cortex*.

[5] Pleger B., Ragert P., Schwenkreis P. (2006). Patterns of cortical reorganization parallel impaired tactile discrimination and pain intensity in complex regional pain syndrome. *Neuroimage*.

[6] Flor H., Elbert T., Knecht S. (1995). Phantom-limb pain as a perceptual correlate of cortical reorganization following arm amputation. *Nature*.

[7] Maihofner C., Handwerker H. O., Neundörfer B., Birklein F. (2003). Patterns of cortical reorganization in complex regional pain syndrome. *Neurology*.

[8] Flor H., Denke C., Schaefer M., Grüsser S. (2001). Effect of sensory discrimination training on cortical reorganisation and phantom limb pain. *The Lancet*.

[9] Moseley G. L., Flor H. (2012). Targeting cortical representations in the treatment of chronic pain. *Neurorehabilitation and Neural Repair*.

[10] Wand B. M., O’Connell N. E., Di Pietro F., Bulsara M. (2011). Managing chronic nonspecific low back pain with a sensorimotor retraining approach: exploratory multiple-baseline study of 3 participants. *Physical Therapy*.

[11] Wand B. M., Abbaszadeh S., Smith A. J., Catley M. J., Moseley G. L. (2013). Acupuncture applied as a sensory discrimination training tool decreases movement-related pain in patients with chronic low back pain more than acupuncture alone: a randomised cross-over experiment. *British Journal of Sports Medicine*.

[12] Diers M., Koeppe C., Diesch E. (2007). Central processing of acute muscle pain in chronic low back pain patients: an EEG mapping study. *Journal of Clinical Neurophysiology*.

[13] Kregel J., Meeus M., Malfliet A. (2015). Structural and functional brain abnormalities in chronic low back pain: a systematic review☆. *Seminars in Arthritis and Rheumatism*.

[14] Nolan M. F. (1985). Quantitative measure of cutaneous sensation. Two-point discrimination values for the face and trunk. *Physical Therapy*.

[15] Moberg E. (1990). Two-point discrimination test. A valuable part of hand surgical rehabilitation, e.g. in tetraplegia. *Scandinavian Journal of Rehabilitation Medicine*.

[16] Adamczyk W. M., Saulicz O., Saulicz E., Luedtke K. (2018). Tactile acuity (dys)function in acute nociceptive low back pain. *Pain*.

[17] Adamczyk W., Sługocka A., Saulicz O., Saulicz E. (2016). The point-to-point test: a new diagnostic tool for measuring lumbar tactile acuity? Inter and intra-examiner reliability study of pain-free subjects. *Manual Therapy*.

[18] Adamczyk W. M., Sługocka A., Mehlich K. (2018). Preliminary validation of a two-point estimation task for the measurement of sensory dissociation in patients with chronic low back pain. *Pain Medicine*.

[19] Harvie D. S., Edmond-Hank G., Smith A. D. (2018). Tactile acuity is reduced in people with chronic neck pain. *Musculoskeletal Science and Practice*.

[20] GBD 2017 Disease and Injury Incidence and Prevalence Collaborators (2018). Global, regional, and national incidence, prevalence, and years lived with disability for 354 diseases and injuries for 195 countries and territories, 1990-2017: a systematic analysis for the Global Burden of Disease Study 2017. *Lancet*.

[21] Hartvigsen J., Hancock M. J., Kongsted A. (2018). What low back pain is and why we need to pay attention. *The Lancet*.

[22] Zingaretti P., Petta A. M., Cruciani G., Spitoni G. F. (2019). Tactile sensitivity, tactile acuity, and affective touch: from childhood to early adolescence. *Somatosensory & Motor Research*.

[23] Brodoehl S., Klingner C., Stieglitz K., Witte O. W. (2013). Age-related changes in the somatosensory processing of tactile stimulation—an fMRI study. *Behavioural Brain Research*.

[24] Woodward K. L. (1993). The relationship between skin compliance, age, gender, and tactile discriminative thresholds in humans. *Somatosensory & Motor Research*.

[25] Pleger B., Wilimzig C., Nicolas V. (2016). A complementary role of intracortical inhibition in age-related tactile degradation and its remodelling in humans. *Scientific Reports*.

[26] Flor H. (2003). Cortical reorganisation and chronic pain: implications for rehabilitation. *Journal of Rehabilitation Medicine*.

[27] Walter S. D., Eliasziw M., Donner A. (1998). Sample size and optimal designs for reliability studies. *Statistics in Medicine*.

[28] Ehrenbrusthoff K., Ryan C. G., Grüneberg C. (2016). The intra- and inter-observer reliability of a novel protocol for two-point discrimination in individuals with chronic low back pain. *Physiological Measurement*.

[29] Catley M. J., Tabor A., Wand B. M., Moseley G. L. (2013). Assessing tactile acuity in rheumatology and musculoskeletal medicine-how reliable are two-point discrimination tests at the neck, hand, back and foot?. *Rheumatology*.

[30] Wand B. M., Di Pietro F., George P., O’Connell N. E. (2010). Tactile thresholds are preserved yet complex sensory function is impaired over the lumbar spine of chronic non-specific low back pain patients: a preliminary investigation. *Physiotherapy*.

[31] Merz O., Wolf U., Robert M., Gesing V., Rominger M. (2013). Validity of palpation techniques for the identification of the spinous process L5. *Manual Therapy*.

[32] Catley M. J., O’Connell N. E., Berryman C., Ayhan F. F., Moseley G. L. (2014). Is tactile acuity altered in people with chronic pain? a systematic review and meta-analysis. *The Journal of Pain*.

[33] Portney L. G., Watkins M. P. (2009). *Foundations of Clinical Research: Applications to Practice*.

[34] Guidetti L., Placentino U., Baldari C. (2017). Reliability and criterion validity of the smartphone inclinometer application to quantify cervical spine mobility. *Clinical Spine Surgery*.

[35] Audette I., Dumas J.-P., Côté J. N., De Serres S. J. (2010). Validity and between-day reliability of the cervical range of motion (CROM) device. *Journal of Orthopaedic & Sports Physical Therapy*.

[36] Lisanti P., Verdisco L. A. (1994). Perceived body space and self-esteem in adult females with chronic low back pain. *Orthopaedic Nursing*.

[37] Moseley L. G. (2008). I can’t find it! Distorted body image and tactile dysfunction in patients with chronic back pain. *Pain*.

[38] Luedtke K., Adamczyk W., Mehrtens K. (2018). Upper cervical two—point discrimination thresholds in migraine patients and headache—free controls. *Journal of Headache and Pain*.

[39] Falling C., Mani R. (2016). Ageing and obesity indices influences the tactile acuity of the low back regions: a cross-sectional study. *Manual Therapy*.

[40] Moseley G. L., Gallagher L., Gallace A. (2012). Neglect-like tactile dysfunction in chronic back pain. *Neurology*.

[41] Adamczyk W. M., Luedtke K., Saulicz O., Saulicz E. (2018). Sensory dissociation in chronic low back pain: two case reports. *Physiotherapy Theory and Practice*.

[42] Morone G., Iosa M., Paolucci T. (2012). Efficacy of perceptive rehabilitation in the treatment of chronic nonspecific low back pain through a new tool: a randomized clinical study. *Clinical Rehabilitation*.

[43] Hohmann C., Ullrich I., Lauche R. (2012). The benefit of a mechanical needle stimulation pad in patients with chronic neck and lower back pain: two randomized controlled pilot studies. *Evidence-Based Complementary and Alternative Medicine*.

[44] Gutknecht M., Mannig A., Waldvogel A., Wand B. M., Luomajoki H. (2015). The effect of motor control and tactile acuity training on patients with non-specific low back pain and movement control impairment. *Journal of Bodywork and Movement Therapies*.

[45] Moberg E. (1958). Objective methods for determining the functional value of sensibility in the hand. *The Journal of Bone and Joint Surgery. British Volume*.

[46] Nolan M. F. (1982). Two-point discrimination assessment in the upper limb in young adult men and women. *Physical Therapy*.

[47] Tong J., Mao O., Goldreich D. (2013). Two-point orientation discrimination versus the traditional two-point test for tactile spatial acuity assessment. *Frontiers in Human Neuroscience*.

